# Comparison of clinicopathologic characteristics, epigenetic biomarkers and prognosis between renal pelvic and ureteral tumors in upper tract urothelial carcinoma

**DOI:** 10.1186/s12894-018-0334-7

**Published:** 2018-03-27

**Authors:** Dong Fang, Shiming He, Gengyan Xiong, Nirmish Singla, Zhenpeng Cao, Lei Zhang, Xuesong Li, Liqun Zhou

**Affiliations:** 1 Department of Urology, Peking University First Hospital, Institute of Urology, Peking University, National Urological Cancer Centre, No. 8 Xishiku St, Xicheng District, Beijing, 100034 China; 2 Department of Urology, University of Texas Southwestern Medical Center, Dallas, TX USA

**Keywords:** Methylation, Prognosis, Radical nephroureterectomy (RNU), Renal pelvis, Upper tract urothelial carcinomas (UTUC), Ureter

## Abstract

**Background:**

There's no consensus about the difference between renal pelvic and ureteral tumors in terms of clinical features, pathological outcomes, epigenetic biomarkers and prognosis.

**Methods:**

The data of 341 patients with renal pelvic tumors and 271 patients with ureteral tumors who underwent radical nephroureterectomy between 1999 and 2011 were retrospectively reviewed. The clinicopathologic features, gene promoters methylation status and oncologic outcomes were compared. Regression analysis was performed to identify oncologic prognosticators.

**Results:**

Patients with ureteral tumors were relatively older (*p* = 0.002), and had higher likelihood of pre-operative renal insufficiency (*p* < 0.001), hypertension (*p* = 0.038) and hydronephrosis (*P* < 0.001), while in patients with renal pelvic tumors gross hematuria was more prevalent (*p* < 0.001). Renal pelvic tumors tended to exhibit non-organ-confined disease (*p* = 0.004) and larger tumor diameter (*p* = 0.001), while ureteral tumors had a higher likelihood of exhibiting high grade (*p* < 0.001) and sessile architecture (*p* = 0.023). Hypermethylated gene promoters were significantly more prevalent in renal pelvic tumors (*p* < 0.001), specifically for TMEFF2, GDF15, RASSF1A, SALL3 and ABCC6 (all *p* < 0.05). Tumor location failed to independently predict cancer-specific survival, overall survival, intravesical or contralateral recurrence (all *p* > 0.05), while gene methylation status was demonstrated to be an independent prognostic factor.

**Conclusion:**

Renal pelvic tumors and ureteral tumors exhibited significant differences in clinicopathologic characteristics and epigenetic biomarkers. Gene promoter methylation might be an important mechanism in explaining distinct tumor patterns and behaviors in UTUC.

## Background

Urothelial carcinomas could be located anywhere throughout the whole urinary tract, e.g. renal pelvis, ureter, bladder and urethra [1]. Upper tract urothelial carcinoma refers to renal pelvic and ureteral tumors [2], with radical nephroureterectomy (RNU) and excision of the bladder cuff as the standard treatment [3].

Since both ureteral tumors and renal pelvic tumors originate from the urothelium, they have been traditionally classified as a single entity (UTUC) and managed in a relatively similar fashion, barring nephron-sparing approaches for more distally located tumors. In recent years there have been studies focusing on the impact of tumor location on prognosis [4–7], though evidence concerning clinical, pathological and genetic differences between renal pelvic and ureteral tumors remains scarce [8].

Microsatellite instability and hypermethylation have been proposed as key genetic differences between bladder cancer and UTUC [9–11], and we recently found gene promoter methylation status to hold biologic and prognostic significance in UTUC [12]. In the present study based on a large cohort of Chinese UTUC patients, we investigated the difference between renal pelvic and ureteral tumors in terms of clinical features, pathological outcomes, epigenetic biomarkers and prognosis.

## Methods

### Patient selection

Review board approve from Peking University First Hospital was acquired and all patients signed the informed consent to participate into the study. We evaluated consecutive Chinese UTUC patients who underwent RNU from 1999 to 2011 at Peking University First Hospital. We excluded patients with synchronous bilateral UTUC, distant metastasis prior to surgery or without complete follow-up data. Patients without available DNA from the surgical specimen for analysis of gene promoter methylation status were also excluded. Six hundred and-twelve patients were finally enrolled for analysis.

RNU including an extravesical excision of distal ureter by open Gibson incision was performed in all patients. No patients received neoadjuvant chemotherapy or prophylactic post-operative intravesical instillation (MMC or THP), while adjuvant chemotherapy for high-risk patients was administered at the treating physician’s discretion.

### Patient evaluation

Computed tomography (CT) or magnetic resonance imaging (MRI), urological ultrasound, and cystoscopy were performed in all patients before surgery. Urinary cytology and ureteroscopy were used to help diagnosis. Renal function was assessed by estimated glomerular filtration rate (eGFR) calculated by Chinese population-specific equation: eGFR(ml/min/1.73m^2^) = 175 × Scr^-1.234^ × age^-0.179^ (× 0.79 if female) [13]. Ipsilateral hydronephrosis was determined pre-operatively.

Patients were categorized into 2 groups (renal pelvis versus ureter) in the current analysis based on the location of the main lesion on pathological specimen (e.g. the highest tumor stage). Pathological examination was performed according to standard procedures by a dedicated pathologist. Tumors were staged per the 2002 Union for International Cancer Control (UICC) TNM classification, and grading was evaluated per the World Health Organization (WHO) classification of 1973.

### DNA extraction and methylation analysis

The procedure of DNA extraction and methylation analysis has been reported in a previous publication by our research group [12]. Based on the formalin-fixed paraffin-embedded tumor samples stored in our center, DNA samples were obtained and were treated for bisulfite transformation. Methylation-sensitive polymerase chain reaction (MSP) was used to analyze the gene promoters methylation status [14]. We used methylated human genomic DNA (Qiagen, Hilden, Germany) as positive control and water blanks with polymerase chain reaction mixtures as negative control. Based on previous literatures we did not detect the methylation status of the gene promoters in matched paracarcinoma tissues due to the limited methylation rates [15–20].

### Follow-up schedule

Follow-up consisted of cystoscopy, chest X-ray, urine cytology, and serum creatinine every 3 months for the first 3 years and then once per year thereafter. Abdominal ultrasound or CT/MRI was performed to examine the contralateral upper urinary tract. Overall survival (OS), cancer specific survival (CSS), bladder recurrence and contralateral recurrence were documented and compared by tumor location. Bladder recurrence was defined as subsequent bladder tumor detected by cystoscopy and confirmed by pathologic examination, and contralateral recurrence was defined as urothelial carcinoma found in the contralateral upper urinary tract. Cause of death was determined by death certificates, by medical notes or by the patients’ responsible physicians.

### Statistical analysis

Statistical analysis was carried by using R software i386 2.15.3 (R Foundation for Statistical Computing, http://www.r-project.org) and SPSS 20.0 (IBM Corp, Armonk, NY, USA). Categorical variables were tested by the Pearson’s test and Chi-square test, while variables with a continuous distribution were evaluated by the Mann-Whitney U test. Cox regression model was used for survival analysis, and Kaplan-Meier curves including log-rank test was employed. A single-sided *p* value of lower than 0.05 was regarded as statistical significance.

## Results

### Clinical characteristics

Overall, 612 patients with either renal pelvic tumor (*n* = 341; 55.7%) or ureteral tumor (*n* = 271; 44.3%) were included. The median age of the entire cohort of patients was 68 (interquartile range, IQR: 60–74) years, and 272 (44.4%) were female, with a male:female ratio of 1.25:1. Previous or concomitant bladder cancer was present in 67 patients (10.9%).

The clinical features are exhibited in Table 1, grouped by tumor location. Patients with ureteral tumors were relatively older (*p* = 0.002), and suffered from high likelihood of pre-operative renal insufficiency (*p* < 0.001), hypertension (*p* = 0.038) and hydronephrosis (*P* < 0.001), while in patients with renal pelvic tumors gross hematuria was more prevalent (*p* < 0.001).

**Table 1:** Clinical and pathological characteristics of all UTUC patients stratified by tumor location

		Tumor location		Univariate analysis	
	All	Renal pelvis	Ureter	Chi-square or Z	*p* value
Patients, no. (%)	612 (100)	341 (55.7)	271 (44.3)		
Pre-operative characteristic					
Gender, no. (%)				0.160	0.743
Male	340 (55.6)	187 (54.8)	153 (56.5)		
Female	272 (44.4)	154 (45.2)	118 (43.5)		
Age, no. (%)				4.929	0.027*
<70	340 (55.6)	203 (59.5)	137 (50.6)		
≥ 70	272 (44.4)	138 (40.5)	134 (49.4)		
Age, mean ± SD		65.29 ± 11.11	68.07 ± 10.20	−3.173	0.002*
Previous or concomitant bladder cancer, no. (%)				1.931	0.193
Absent	545 (89.1)	309 (90.6)	236 (87.1)		
Present	67 (10.9)	32 (9.4)	35 (12.9)		
Initial complaint, no. (%)				24.205	< 0.001*
Absent	84 (13.7)	26 (7.6)	58 (21.4)		
Present	528 (86.3)	315 (92.4)	213 (78.6)		
Gross hematuria, no. (%)				65.132	< 0.001*
Absent	148 (24.2)	40 (11.7)	108 (39.9)		
Present	464 (75.8)	301 (88.3)	163 (60.1)		
Preoperative renal function, no. (%)				23.703	< 0.001*
End-stage CKD (eGFR<15)	34 (5.6)	24 (7.0)	10 (3.7)		
Moderate CKD (60>eGFR≥15)	198 (32.4)	83 (24.3)	115 (42.4)		
Early CKD (eGFR≥60)	378 (61.8)	233 (68.3)	145 (53.5)		
eGFR, mean ± SD		69.69 ± 30.11	62.43 ± 22.32	−4.329	< 0.001*
Side, no. (%)				1.115	0.329
Left	315 (51.5)	182 (53.4)	133 (49.1)		
Right	297 (48.5)	159 (46.6)	138 (50.9)		
Hydronephrosis, no. (%)				134.680	< 0.001*
Absent	273 (44.6)	223 (65.4)	50 (18.5)		
Present	339 (55.4)	118 (34.6)	221 (81.5)		
Multifocality, no. (%)				0.339	0.563
Single	472 (77.1)	266 (78.0)	206 (76.0)		
Multiple	140 (22.9)	75 (22.0)	65 (24.0)		
Smoking, no. (%)				0.050	0.836
No	497 (81.2)	278 (81.5)	219 (80.8)		
Yes	115 (18.8)	63 (18.5)	52 (19.2)		
Alcohol, no. (%)				0.697	0.452
No	539 (88.1)	297 (87.1)	242 (89.3)		
Yes	73 (11.9)	44 (12.9)	29 (10.7)		
Diabetes, no. (%)				0.249	0.661
No	511 (83.5)	287 (84.2)	224 (82.7)		
Yes	101 (16.5)	54 (15.8)	47 (17.3)		
Hypertension, no. (%)				4.454	0.038*
No	363 (59.3)	215 (63.0)	148 (54.6)		
Yes	249 (40.7)	126 (37.0)	123 (45.4)		
Pre-RNU ureteroscopy, no. (%)				20.495	< 0.001*
No	536 (87.6)	317 (93.0)	219 (80.8)		
Yes	76 (12.4)	24 (7.0)	52 (19.2)		
Pathological outcomes					
Architecture, no. (%)				40.135	< 0.001*
Papillary	479 (78.3)	299 (87.7)	180 (66.4)		
Sessile	133 (21.7)	42 (12.3)	91 (33.6)		
Tumor stage, no. (%)				0.094	0.796
Ta-T1	206 (33.7)	113 (33.1)	93 (34.3)		
T2–4	406 (66.3)	228 (66.9)	178 (65.7)		
Tumor grade, no. (%)				31.628	< 0.001*
G1	19 (3.1)	4 (1.2)	15 (5.5)		
G2	334 (54.6)	218 (63.9)	116 (42.8)		
G3	259 (42.3)	119 (34.9)	140 (51.7)		
Lymph node status, no. (%)				4.014	0.051
N0 or Nx	571 (93.3)	312 (91.5)	259 (95.6)		
N+	41 (6.7)	29 (8.5)	12 (4.4)		
Non-organ-confined disease, no. (%)				8.257	0.004*
No	412 (67.3)	213 (62.5)	199 (73.4)		
Yes	200 (32.7)	128 (37.5)	72 (26.6)		
Tumor size, mean ± SD		3.58 ± 2.15	3.27 ± 2.41	−3.342	0.001*
Histologic Subtype					
Tumor necrosis, no. (%)				0.038	0.901
No	537 (87.7)	300 (88.0)	237 (87.5)		
Yes	75 (12.3)	41 (12.0)	34 (12.5)		
Squamous metaplasia, no. (%)				0.038	0.878
No	566 (92.5)	316 (92.7)	250 (92.3)		
Yes	46 (7.5)	25 (7.3)	21 (7.7)		
Sarcomatoid metaplasia, no. (%)				0.039	0.843
No	586 (95.8)	327 (95.9)	259 (95.6)		
Yes	26 (4.2)	14 (4.1)	12 (4.4)		
Gland-like differentiation, no. (%)				2.738	0.119
No	591 (96.6)	333(97.7)	258 (95.2)		
Yes	21 (3.4)	8(2.3)	13 (4.8)		
Presence of CIS, no. (%)				3.987	0.071
No	596 (97.4)	336 (98.5)	260 (95.9)		
Yes	16 (2.6)	5 (1.5)	11 (4.1)		

*UTUC* upper tract urothelial carcinoma, *CKD* chronic kidney disease, *eGFR* estimated glomerular filtration rate, *RNU* radical nephroureterectomy, *CIS* carcinoma in situ, *SD* standard deviation, *HR* Hazard Ratio, *CI* confidence interval

*Statistically significant

### Pathological outcomes

The frequencies of muscle-invasive disease (≥pT2) and lymph node metastasis were comparable between groups; however, non-organ-confined tumors (≥pT3) were more prevalent in patients with renal pelvic tumors versus the ureteral tumor counterparts (*p* = 0.004). In concordance with this observation, sessile architecture and larger tumor size were more prevalent in patients with renal pelvic tumors as well (*p* < 0.001). G3 tumor grade, however, was present more often in ureteral tumors (*p* < 0.001). There were no differences in terms of squamous and glandular differentiation.

### Molecular biomarkers

**Fig. 1:**
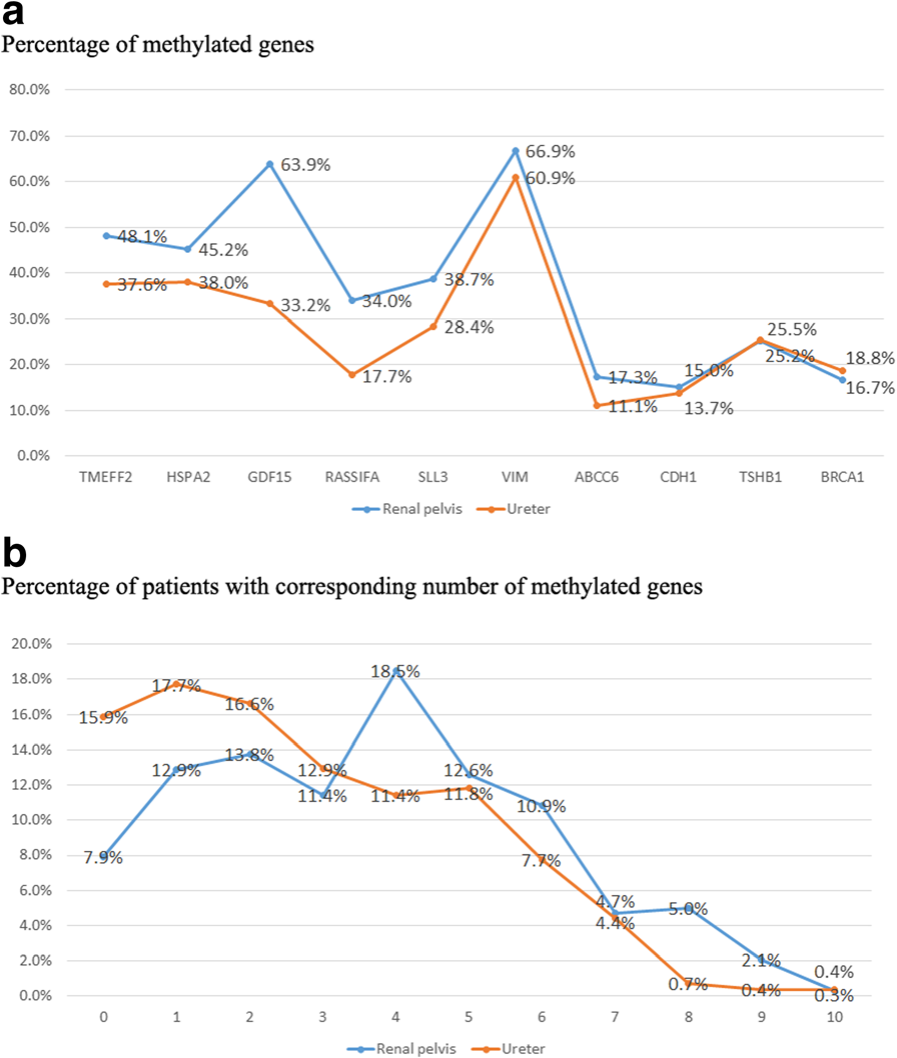
Distribution of aberrant methylated genes in renal pelvic and ureteral tumors (**a**) and the cumulative numbers of aberrant methylated genes (**b**)

In 542 patients (88.6%) at least one methylated gene promoter was found, with a mean methylated genes number of 3.33 ± 2.31. Methylation was present significantly more frequently in renal pelvic tumors (Table 2), particularly with a higher rate of methylated TMEFF2, GDF15, RASSF1A, SALL3 and ABCC6 (all *p* < 0.05) (Fig. 1a). The mean number methylated genes in renal pelvic tumors was 3.71 ± 2.33, while in ureteral tumors was only 2.85 ± 2.19 (*p* < 0.001). Besides many patients with ureteral tumors presented with only very few methylated genes. (Fig. 1b).

**Table 2:** Molecular biomarkers

	All	Renal pelvis	Ureter	Chi-square or Z	*p* value
Patients, no. (%)	612 (100)	341 (55.7)	271 (44.3)		
TMEFF2, no. (%)				6.717	0.011*
Unmethylated	346 (56.5)	177 (51.9)	169 (62.4)		
Methylated	266 (43.5)	164 (48.1)	102 (37.6)		
HSPA2, no. (%)				3.172	0.083
Unmethylated	355 (58.0)	187 (54.8)	168 (62.0)		
Methylated	257 (42.0)	154 (45.2)	103 (38.0)		
GDF15, no. (%)				57.000	< 0.001*
Unmethylated	304 (49.7)	123 (36.1)	181 (66.8)		
Methylated	308 (50.3)	218 (63.9)	90 (33.2)		
RASSF1A, no. (%)				20.465	< 0.001*
Unmethylated	448 (73.2)	225 (66.0)	223 (82.3)		
Methylated	164 (26.8)	116 (34.0)	48 (17.7)		
SALL3, no. (%)				7.119	0.008*
Unmethylated	403 (65.8)	209 (61.3)	194 (71.6)		
Methylated	209 (34.2)	132 (38.7)	77 (28.4)		
VIM, no. (%)				2.347	0.128
Unmethylated	219 (35.8)	113 (33.1)	106 (39.1)		
Methylated	393 (64.2)	228 (66.9)	165 (60.9)		
ABCC6, no. (%)				4.719	0.037*
Unmethylated	523 (85.5)	282 (82.7)	241 (88.9)		
Methylated	89 (14.5)	59 (17.3)	30 (11.1)		
CDH1, no. (%)				0.208	0.728
Unmethylated	524 (85.6)	290 (85.0)	234 (86.3)		
Methylated	88 (14.4)	51 (15.0)	37 (13.7)		
THBS1, no. (%)				0.005	1.000
Unmethylated	457 (74.7)	255 (74.8)	202 (74.5)		
Methylated	155 (25.3)	86 (25.2)	69 (25.5)		
BRCA1, no. (%)				0.460	0.523
Unmethylated	504 (82.4)	284 (83.3)	220 (81.2)		
Methylated	108 (17.6)	57 (16.7)	51 (18.8)		
Presence of hypermethylation in any gene, no. (%)				9.420	0.003*
Unmethylated	70 (11.4)	27 (7.9)	43 (15.9)		
Methylated	542 (88.6)	314 (92.1)	228 (84.1)		
Mean methylated genes		3.71 ± 2.33	2.85 ± 2.19	−4.503	< 0.001*
Number of methylated genes, no. (%)				17.202	< 0.001*
0–2	254 (41.5)	118 (34.6)	136 (50.2)		
3–5	243 (39.7)	145 (42.5)	98 (36.2)		
6–10	115 (18.8)	78 (22.9)	37 (13.7)		
Number of methylated genes, no. (%) in Ta-1				11.251	0.004*
All	206 (100)	113 (54.9)	93 (45.1)		
0–2	95 (46.1)	41 (36.3)	54 (58.1)		
3–5	80 (38.8)	49 (43.4)	31 (33.3)		
6–10	31 (15.0)	23 (20.4)	8 (8.6)		
Number of methylated genes, no. (%) in T2–4				7.318	0.026*
All	406 (100)	228 (56.2)	178 (43.8)		
0–2	159 (39.2)	77 (33.8)	82 (46.1)		
3–5	163 (40.1)	96 (42.1)	67 (37.6)		
6–10	84 (20.7)	55 (24.1)	29 (16.3)		
Number of methylated genes, no. (%) in G1–2				18.433	< 0.001*
All	353 (100)	222 (62.9)	131 (37.1)		
0–2	156 (44.2)	80 (36.0)	76 (58.0)		
3–5	141 (39.9)	97 (43.7)	44 (33.6)		
6–10	56 (15.9)	45 (20.3)	11 (8.4)		
Number of methylated genes, no. (%) in G3				4.449	0.108
All	259 (100)	119 (45.9)	140 (54.1)		
0–2	98 (37.8)	38 (31.9)	60 (42.9)		
3–5	102 (39.4)	48 (40.3)	54 (38.6)		
6–10	59 (22.8)	33 (27.7)	26 (18.6)		

*Statistically significant

*UTUC* upper tract urothelial carcinoma

In subgroup analysis based on tumor stage, renal pelvic tumors exhibited more methylated genes both in non-muscle-invasive and muscle-invasive diseases, while in subgroup analysis based on tumor grade, the difference was significant only in lower tumor stages (G1–2).

### Oncologic outcomes

The median follow-up duration was 64 months. In all 210 (34.3%) patients died and 187 (30.6%) died secondary to urothelial cancer. The cumulative 5-year OS and CSS rates were 69.1% and 71.4%, respectively. Bladder recurrence was found in 174 (28.4%) patients, and 32 (5.2%) patients experienced contralateral recurrence.

**Fig. 2:**
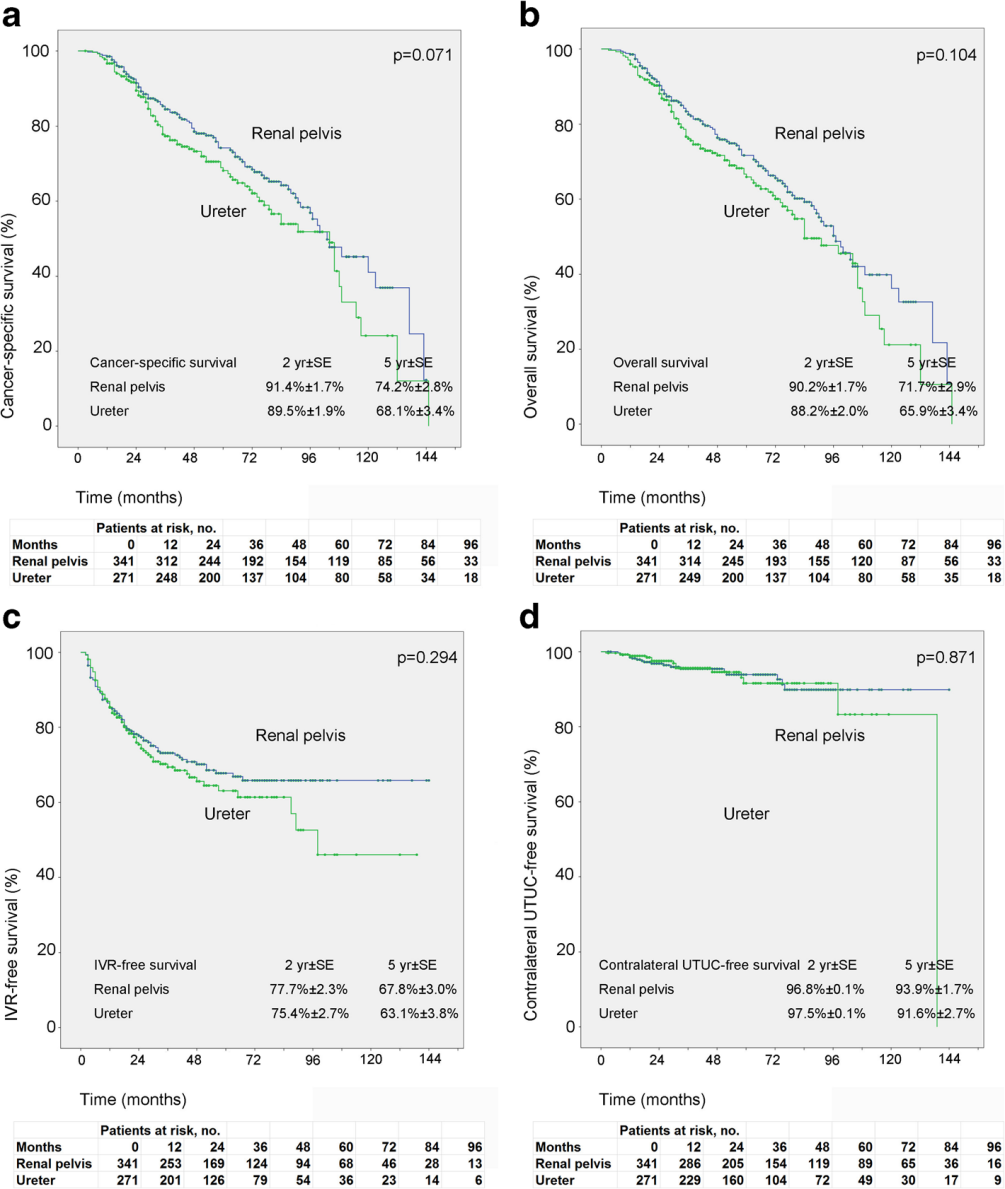
Estimated Kaplan-Meier overall survival (**a**) (*p* = 0.104), cancer specific survival (**b**) (*p* = 0.071), bladder recurrence-free survival (**c**) (*p* = 0.294) and contralateral carcinoma-free survival (**d**) (*p* = 0.871) curves stratified by tumor location

By univariate analysis, there’s no relationship between tumor location (renal pelvis versus ureter) OS (*p* = 0.104), CSS (*p* = 0.071), bladder recurrence (*p* = 0.294) or contralateral recurrence (*p* = 0.871). (Fig. 2).

**Fig. 3:**
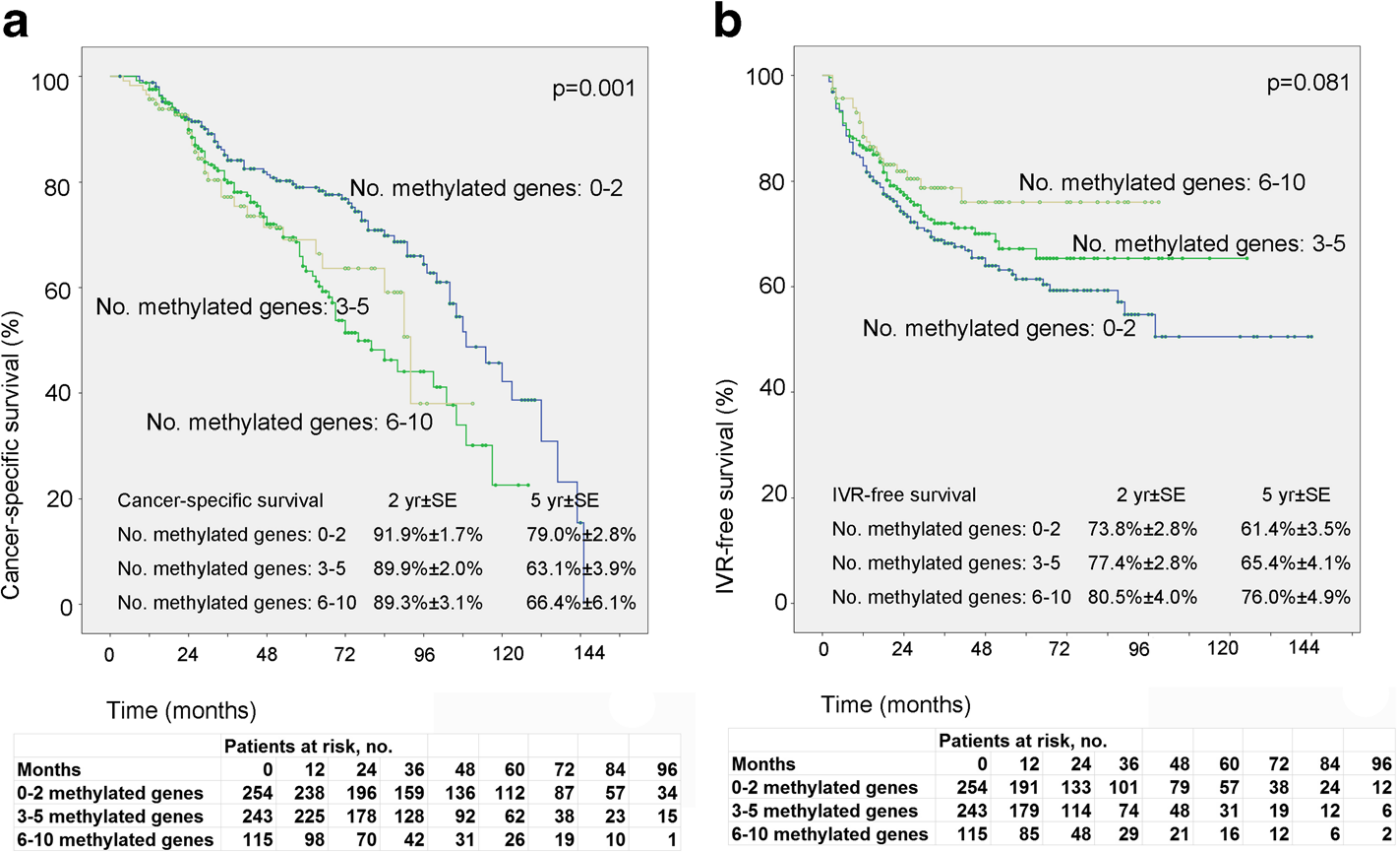
Estimated Kaplan-Meier cancer specific survival (**a**) (*p* = 0.001) and bladder recurrence-free survival (**b**) (*p* = 0.081) curves stratified by numbers of methylated genes (0–2 versus 3–5 versus 6–10)

Other factors, including tumor stage, presence of hydronephrosis, and the methylation status of several genes were proved to be important predictive factors for survival. (Table 3). On Kaplan-Meier analysis, less cumulative number of methylated genes was correlated with better CSS, with mean CSS time of 101 months, 79 months and 77 months for patients with 0–2, 3–5 and 6–10 methylated genes, respectively (Fig. 3a). Though not statistically significant, a trend to higher risk for bladder recurrence in patients with less number of methylated genes (*p* = 0.081, Fig. 3b) was found. Besides the number of methylated genes (as continuous) was found to affect CSS (HR = 1.348, *p* = 0.003) and bladder recurrence (HR = 0.787, *p* = 0.026) in univariate analysis (Table 3 and 4).

**Table 3:** Prognostic factors for cancer-specific survival in the entire cohort of UTUC patients and stratified by tumor location

Variables	All patients (*n* = 612)						Renal pelvis (*n* = 341)						Ureter (*n* = 271)					
	UVA			MVA			UVA			MVA			UVA			MVA		
	HR	95%CI	*p* value	HR	95%CI	*p* value	HR	95%CI	*p* value	HR	95%CI	*p* value	HR	95%CI	*p* value	HR	95%CI	*p* value
Location (ureter vs renal pelvis)	1.302	0.976–1.738	0.073															
Gender (men vs women)	1.611	1.206–2.152	0.001*	1.45	1.07–1.96	0.016*	1.680	1.117–2.527	0.013*	1.46	0.94–2.27	0.091	1.574	1.039–2.383	0.032*	1.43	0.93–2.21	0.103
Age (continuous)	1.020	1.005–1.035	0.009*	1.32	1.07–1.64	0.010*	1.008	0.988–1.028	0.422				1.032	1.008–1.056	0.008*	1.09	0.78–1.52	0.628
Preoperative hydronephosis	1.595	1.183–2.151	0.002*	1.77	1.28–2.45	0.001*	1.766	1.168–2.671	0.007*	1.89	1.20–2.98	0.006*	1.191	0.691–2.053	0.529			
Multifocality (presence of multiple foci vs absence)	1.414	1.028–1.943	0.033*	1.57	1.10–2.24	0.014*	1.675	1.088–2.578	0.019*	1.70	1.03–2.82	0.040*	1.145	0.711–1.845	0.577			
Preoperative renal function(eGFR, continuous)	0.996	0.991–1.001	0.119				1.000	0.993–1.007	0.993				0.989	0.979–0.998	0.021*	0.77	0.55–1.06	0.106
Previous or concomitant BT (presence vs absence)	1.544	1.019–2.339	0.041*	1.53	0.97–2.41	0.070	1.980	1.099–3.568	0.023*	2.03	1.04–3.94	0.037*	1.183	0.657–2.132	0.575			
Gross hematuria (presence vs absence)	0.913	0.650–1.282	0.599				0.625	0.353–1.104	0.105				1.329	0.845–2.090	0.218			
Smoke (presence vs absence)	1.102	0.765–1.588	0.601				1.242	0.757–2.037	0.391				0.985	0.572–1.698	0.958			
Alcohol (presence vs absence)	1.108	0.726–1.690	0.634				1.546	0.912–2.619	0.105				0.713	0.344–1.477	0.363			
Diabetes (presence vs absence)	0.891	0.592–1.340	0.579				0.994	0.563–1.756	0.984				0.797	0.442–1.437	0.451			
Hypertension (presence vs absence)	1.180	0.879–1.584	0.270				1.056	0.689–1.618	0.802				1.230	0.810–1.868	0.332			
Preoperative ureteroscopy	0.616	0.383–0.992	0.046*	0.72	0.44–1.18	0.194	0.234	0.058–0.951	0.042*	0.24	0.06–1.03	0.055	0.663	0.387–1.137	0.135			
Tumor stage^^^ (T4 vs T3 vs T2 vs T1 vs Ta)	1.725	1.443–2.061	< 0.001*	2.42	1.56–3.76	< 0.001*	1.514	1.197–1.915	0.001*	1.83	1.04–3.21	0.035*	2.288	1.716–3.050	< 0.001*	2.53	1.46–4.38	0.001*
Tumor grade^^^ (G3 vs G2 vs G1)	1.593	1.222–2.075	0.001*	0.69	0.36–1.34	0.274	1.284	0.868–1.900	0.211				1.856	1.271–2.710	0.001*	1.30	0.47–3.61	0.611
Lymph node status (N+ vs Nx vs N-)	2.524	1.583–4.023	< 0.001*	1.82	1.08–3.07	0.024*	2.863	1.615–5.074	< 0.001*	2.49	1.26–4.92	0.009*	2.356	1.024–5.417	0.044*	1.18	0.49–2.84	0.713
Architecture (presence of sessile vs absence)	1.974	1.437–2.713	< 0.001*	1.38	0.92–2.07	0.125	2.105	1.242–3.566	0.006*	1.20	0.63–2.28	0.584	1.811	1.186–2.766	0.006*	0.98	0.55–1.75	0.951
CIS (presence of sessile vs absence)	1.027	0.480–2.202	0.994				1.386	0.424–4.535	0.590				0.808	0.296–2.210	0.678			
Necrosis(presence vs absence)	1.925	1.302–2.846	0.001*	1.36	0.84–2.18	0.207	1.606	0.905–2.850	0.105				2.352	1.375–4.025	0.002*	1.36	0.69–2.70	0.376
Squamous metaplasia (presence vs absence)	1.783	1.081–2.943	0.024*	1.45	0.85–2.48	0.171	2.123	1.063–4.241	0.033*	2.31	1.06–5.02	0.034*	1.485	0.716–3.079	0.288			
Sarcomatoid metaplasia (presence vs absence)	2.595	1.526–4.413	< 0.001*	0.79	0.40–1.56	0.493	2.541	1.171–5.513	0.018*	1.03	0.40–2.62	0.955	2.629	1.266–5.459	0.010*	1.00	0.41–2.45	0.993
Gland-like differentiation (presence vs absence)	1.963	0.965–3.995	0.063				3.394	1.239–9.296	0.017*	2.08	0.68–6.34	0.197	1.229	0.449–3.362	0.688			
Tumor size (continuous)	1.172	1.112–1.236	< 0.001*	1.17	1.04–1.32	0.010*	1.163	1.070–1.264	< 0.001*	1.16	0.93–1.44	0.188	1.184	1.110–1.264	< 0.001*	1.26	1.06–1.49	0.008*
TMEFF2 (methylated vs unmethylated)	1.812	1.353–2.427	< 0.001*	1.67	1.12–2.50	0.012*	1.634	1.085–2.459	0.019*	1.16	0.70–1.92	0.562	2.189	1.434–3.340	< 0.001*	1.84	0.97–3.50	0.061
HSPA2 (methylated vs unmethylated)	1.815	1.349–2.442	< 0.001*	1.52	1.03–2.24	0.036*	2.064	1.365–3.119	0.001*	1.40	0.86–2.28	0.180	1.698	1.097–2.626	0.017*	1.08	0.60–1.97	0.793
GDF15 (methylated vs unmethylated)	1.242	0.930–1.660	0.142				1.575	1.025–2.421	0.038*	1.24	0.73–2.12	0.426	1.152	0.741–1.793	0.530			
RASSF1A (methylated vs unmethylated)	1.383	1.002–1.908	0.049*	1.15	0.78–1.70	0.477	1.271	0.824–1.961	0.279				1.796	1.102–2.929	0.019*	1.57	0.87–2.82	0.135
SALL3 (methylated vs unmethylated)	1.214	0.887–1.662	0.226				0.887	0.565–1.392	0.602				1.853	1.190–2.885	0.006*	1.58	0.93–2.68	0.094
VIM (methylated vs unmethylated)	1.360	1.002–1.847	0.049*	0.99	0.68–1.44	0.941	1.630	1.041–2.550	0.033*	1.37	0.81–2.32	0.243	1.208	0.786–1.857	0.388			
ABCC6 (methylated vs unmethylated)	1.430	0.928–2.203	0.105				1.206	0.682–2.134	0.519				2.283	1.165–4.476	0.016*	1.51	0.67–3.38	0.317
CDH1 (methylated vs unmethylated)	1.178	0.766–1.812	0.456				1.112	0.618–2.001	0.724				1.401	0.741–2.649	0.300			
THBS1 (methylated vs unmethylated)	1.131	0.811–1.577	0.468				0.877	0.534–1.439	0.603				1.415	0.899–2.227	0.133			
BRCA1 (methylated vs unmethylated)	0.851	0.565–1.280	0.438				0.678	0.361–1.272	0.226				1.026	0.596–1.765	0.927			
No. methylated genes (continuous)	1.348	1.107–1.641	0.003*	0.62	0.30–1.28	0.193	1.225	0.930–1.613	0.149				1.646	1.234–2.196	0.001*	0.58	0.18–1.82	0.351

*UVA* univariate analysis, *MVA* multivariate analysis, *eGFR* estimated glomerular filtration rate, *UTUC* upper tract urothelial carcinoma, *BT* bladder tumor, *CIS* carcinoma in situ, *HR* Hazard Ratio, *CI* confidence interval

*Statistically significant

**Table 4:** Prognostic factors for bladder recurrence in the entire cohort of UTUC patients and stratified by tumor location

Variables	All patients (*n* = 612)						Renal pelvis (*n* = 341)						Ureter (*n* = 271)					
	UVA			MVA			UVA			MVA			UVA			MVA		
	HR	95%CI	*p* value	HR	95%CI	*p* value	HR	95%CI	*p* value	HR	95%CI	*p* value	HR	95%CI	*p* value	HR	95%CI	*p* value
Location (ureter vs renal pelvis)	1.172	0.870–1.579	0.297															
Gender (men vs women)	1.150	0.854–1.550	0.357				1.279	0.850–1.924	0.239				1.035	0.668–1.606	0.876			
Age (continuous)	0.987	0.974–1.000	0.050				0.983	0.966–1.000	0.055				0.989	0.970–1.010	0.989			
Preoperative hydronephosis	1.173	0.868–1.585	0.298				1.141	0.745–1.748	0.545				1.050	0.608–1.815	0.861			
Multifocality (presence of multiple foci vs absence)	1.732	1.260–2.381	0.001*	1.42	1.01–2.01	0.045*	1.833	1.185–2.835	0.006*	1.75	1.13–2.72	0.012*	1.610	1.011–2.566	0.045*	1.44	0.86–2.43	0.167
Preoperative renal function(eGFR, continuous)	0.999	0.993–1.004	0.671				0.999	0.993–1.006	0.876				0.999	0.989–1.009	0.887			
Previous or concomitant BT (presence vs absence)	1.900	1.267–2.850	0.002*	1.47	0.95–2.28	0.081	1.710	0.931–3.138	0.084				2.034	1.176–3.517	0.011*	1.39	0.75–2.58	0.293
Gross hematuria (presence vs absence)	1.108	0.773–1.588	0.577				1.102	0.572–2.125	0.772				1.268	0.802–2.005	0.310			
Smoke (presence vs absence)	0.946	0.644–1.388	0.775				1.097	0.655–1.836	0.726				0.784	0.441–1.394	0.407			
Alcohol (presence vs absence)	0.771	0.473–1.257	0.297				0.901	0.480–1.692	0.746				0.622	0.286–1.351	0.230			
Diabetes (presence vs absence)	1.086	0.736–1.601	0.679				0.842	0.469–1.515	0.567				1.414	0.837–2.388	0.196			
Hypertension (presence vs absence)	0.774	0.566–1.059	0.109				0.916	0.595–1.412	0.692				0.614	0.391–0.967	0.035*	0.71	0.44–1.15	0.163
Preoperative ureteroscopy	1.631	1.111–2.395	0.012*	1.25	0.83–1.87	0.285	2.087	1.137–3.829	0.018*	1.62	0.87–3.02	0.126	1.319	0.794–2.189	0.285			
Tumor stage^^^ (T4 vs T3 vs T2 vs T1 vs Ta)	0.861	0.731–1.014	0.074				0.956	0.772–1.185	0.683				0.744	0.574–0.965	0.026*	0.81	0.54–1.20	0.292
Tumor grade^^^ (G3 vs G2 vs G1)	0.655	0.504–0.851	0.002*	0.54	0.31–0.93	0.027*	0.515	0.336–0.789	0.002*	0.30	0.13–0.71	0.006*	0.777	0.555–1.089	0.143			
Lymph node status (N+ vs Nx vs N-)	0.326	0.121–0.879	0.027*	0.45	0.17–1.23	0.120	0.491	0.180–1.338	0.164				0.046	0.000–5.017	0.199			
Architecture (presence of sessile vs absence)	0.718	0.479–1.075	0.108				0.721	0.349–1.490	0.377				0.644	0.389–1.067	0.087			
CIS (presence of sessile vs absence)	1.604	0.789–3.262	0.192				1.498	0.369–6.086	0.572				1.555	0.676–3.573	0.299			
Necrosis(presence vs absence)	1.164	0.750–1.806	0.498				1.532	0.881–2.664	0.131				0.810	0.390–1.682	0.572			
Squamous metaplasia(presence vs absence)	0.626	0.308–1.274	0.196				0.630	0.231–1.720	0.367				0.609	0.223–1.665	0.334			
Sarcomatoid metaplasia (presence vs absence)	0.490	0.182–1.321	0.159				0.465	0.114–1.887	0.284				0.541	0.133–2.204	0.392			
Gland-like differentiation (presence vs absence)	0.576	0.184–1.806	0.344				0.619	0.086–4.452	0.634				0.509	0.125–2.073	0.346			
Tumor size (continuous)	0.920	0.850–0.996	0.039*	0.91	0.78–1.06	0.213	0.967	0.872–1.072	0.520				0.875	0.771–0.991	0.036*	0.83	0.64–1.08	0.170
TMEFF2 (methylated vs unmethylated)	0.714	0.521–0.978	0.036*	0.91	0.60–1.38	0.657	0.839	0.554–1.268	0.404				0.593	0.358–0.984	0.043*	1.00	0.50–2.00	0.999
HSPA2 (methylated vs unmethylated)	0.704	0.511–0.968	0.031*	0.82	0.55–1.24	0.348	0.792	0.519–1.207	0.278				0.626	0.380–1.033	0.067			
GDF15 (methylated vs unmethylated)	0.823	0.611–1.110	0.203				0.936	0.616–1.421	0.755				0.738	0.456–1.196	0.217			
RASSF1A (methylated vs unmethylated)	0.598	0.407–0.878	0.009*	0.69	0.45–1.07	0.095	0.786	0.499–1.237	0.298				0.318	0.138–0.731	0.007*	0.41	0.17–0.97	0.042*
SALL3 (methylated vs unmethylated)	0.725	0.519–1.013	0.059				0.669	0.427–1.048	0.079				0.837	0.505–1.386	0.489			
VIM (methylated vs unmethylated)	0.862	0.636–1.167	0.336				1.302	0.831–2.041	0.249				0.567	0.367–0.875	0.010*	0.64	0.38–1.06	0.081
ABCC6 (methylated vs unmethylated)	0.805	0.499–1.297	0.373				1.007	0.578–1.752	0.981				0.508	0.185–1.392	0.188			
CDH1 (methylated vs unmethylated)	0.681	0.413–1.124	0.133				0.870	0.474–1.597	0.654				0.466	0.188–1.155	0.099			
THBS1 (methylated vs unmethylated)	0.960	0.678–1.359	0.818				1.044	0.651–1.675	0.858				0.872	0.522–1.457	0.601			
BRCA1 (methylated vs unmethylated)	0.977	0.659–1.448	0.908				0.858	0.485–1.515	0.597				1.131	0.655–1.955	0.659			
No. methylated genes(continuous)	0.787	0.637–0.972	0.026*	1.14	0.56–2.34	0.718	0.906	0.688–1.193	0.483				0.656	0.460–0.935	0.020*	0.96	0.34–2.72	0.934

*Statistically significant

*UVA* univariate analysis, *MVA* multivariate analysis, *eGFR* estimated glomerular filtration rate, *UTUC* upper tract urothelial carcinoma, *BT* bladder tumor, *CIS* carcinoma in situ, *HR* Hazard Ratio, *CI* confidence interval

Sub-group analysis demonstrated differences in oncologic prognosticators for CSS and bladder recurrence based on tumor location (Table 3 and 4). Rerunning the dataset by dividing patients into renal pelvic tumors only (*n* = 304), ureteral tumors only (*n* = 267) and both renal pelvic and ureteral tumors (*n* = 41) did not change the results (Table 5).

**Table 5:** Comparison in patients with renal pelvis tumor only and with ureteral tumor only

	Location				Comparison between three groups		Comparison after excluding cases in both locations	
	All	Renal pelvis only	Ureter only	Both locations	Chi-square or Z	*p* value	Chi-square or Z	*p* value
Patients, no. (%)	612 (100)	304 (49.7)	267 (43.6)	41 (6.7)				
Pre-operative characteristic								
Gender, no. (%)					1.595	0.450	0.495	0.501
Male	340 (55.6)	163 (53.6)	151 (56.6)	26 (63.4)				
Female	272 (44.4)	141 (46.4)	116 (43.4)	15 (36.6)				
Age, no. (%)					5.554	0.062	5.391	0.023*
<70	340 (55.6)	182 (59.9)	134 (50.2)	24 (58.5)				
≥ 70	272 (44.4)	122 (40.1)	133 (49.8)	17 (41.5)				
Age, mean ± SD		65.09 ± 11.32	68.12 ± 10.22	66.52 ± 10.79	11.059	0.004*	−3.298	0.001*
Previous or concomitant bladder cancer, no. (%)					31.791	< 0.001*	8.721	0.004*
Absent	545 (89.1)	286 (94.1)	232 (86.9)	27 (65.9)				
Present	67 (10.9)	18 (5.9)	35 (13.1)	14 (34.1)				
Initial complaint, no. (%)					23.992	< 0.001*	23.745	< 0.001*
Absent	84 (13.7)	22 (7.2)	57 (21.3)	5 (12.2)				
Present	528 (86.3)	282 (92.8)	210 (78.7)	36 (87.8)				
Gross hematuria, no. (%)					66.717	< 0.001*	65.579	< 0.001*
Absent	148 (24.2)	33 (10.9)	107 (40.1)	8 (19.5)				
Present	464 (75.8)	271 (89.1)	160 (59.9)	33 (80.5)				
Preoperative renal function, no. (%)					39.081	< 0.001*	29.841	< 0.001*
End-stage CKD (eGFR<15)	34 (5.6)	21 (6.9)	10 (3.7)	3 (7.3)				
Moderate CKD (60>eGFR≥15)	198 (32.4)	64 (21.1)	112 (41.9)	22 (53.7)				
Early CKD (eGFR≥60)	378 (61.8)	218 (71.7)	144 (53.9)	16 (39.0)				
eGFR, mean ± SD		71.30 ± 29.38	62.63 ± 22.32	55.80 ± 31.99	34.160	< 0.001*	−5.108	< 0.001*
Hydronephrosis, no. (%)					156.085	< 0.001*	151.247	< 0.001*
Absent	273 (44.6)	212 (69.7)	49 (18.4)	12 (29.3)				
Present	339 (55.4)	92 (30.3)	218 (81.6)	29 (70.7)				
Multifocality, no. (%)					156.779	< 0.001*	10.618	< 0.001*
Single	472 (77.1)	266 (87.5)	206 (77.2)	0				
Multiple	140 (22.9)	38 (12.5)	61 (22.8)	41 (100)				
Pathological outcomes								
Architecture, no. (%)					39.792	< 0.001*	39.811	< 0.001*
Papillary	479 (78.3)	269 (88.5)	178 (66.7)	32 (78.0)				
Sessile	133 (21.7)	35 (12.5)	89 (33.3)	9 (22.0)				
Tumor stage, no. (%)					0.160	0.923	0.155	0.723
Ta-T1	206 (33.7)	100 (32.9)	92 (34.5)	14 (34.1)				
T2–4	406 (66.3)	204 (67.1)	175 (65.5)	27 (65.9)				
Tumor grade, no. (%)					30.572	< 0.001*	28.242	< 0.001*
G1	19 (3.1)	4 (1.3)	15 (5.6)	0				
G2	334 (54.6)	214 (70.4)	115 (43.1)	25 (61.0)				
G3	259 (42.3)	106 (34.9)	137 (51.3)	16 (39.0)				
Lymph node status, no. (%)					3.772	0.152	3.769	0.064
N0 or Nx	571 (93.3)	278 (91.4)	255 (95.5)	38 (92.7)				
N+	41 (6.7)	26 (8.6)	12 (4.5)	3 (7.3)				
Non-organ-confined disease, no. (%)					10.339	0.006*	9.592	0.002*
No	412 (67.3)	186 (61.2)	196 (73.4)	30 (73.2)				
Yes	200 (32.7)	118 (38.8)	71 (26.6)	11 (26.8)				
Tumor size, mean ± SD		3.56 ± 1.94	3.25 ± 2.40	3.89 ± 3.39	13.014	0.001*	−3.695	< 0.001*
Methylation status								
TMEFF2, no. (%)					6.972	0.031*	6.481	0.011*
Unmethylated	346 (56.5)	158 (52.0)	167 (62.5)	21 (51.2)				
Methylated	266 (43.5)	146 (48.0)	100 (37.5)	20 (48.8)				
HSPA2, no. (%)					3.398	0.183	3.064	0.089
Unmethylated	355 (58.0)	167 (54.9)	166 (62.2)	22 (53.7)				
Methylated	257 (42.0)	137 (45.1)	101 (37.8)	19 (46.3)				
GDF15, no. (%)					56.507	< 0.001*	56.310	< 0.001*
Unmethylated	304 (49.7)	107 (35.2)	178 (66.7)	19 (46.3)				
Methylated	308 (50.3)	197 (64.8)	89 (33.3)	22 (53.7)				
RASSF1A, no. (%)					22.562	< 0.001*	22.341	< 0.001*
Unmethylated	448 (73.2)	197 (64.8)	220 (82.4)	31 (75.6)				
Methylated	164 (26.8)	107 (35.2)	47 (17.6)	10 (24.4)				
SALL3, no. (%)					9.797	0.007*	6.982	0.010*
Unmethylated	403 (65.8)	188 (61.8)	193 (72.3)	22 (53.7)				
Methylated	209 (34.2)	116 (38.2)	74 (27.7)	19 (46.3)				
VIM, no. (%)					3.367	0.186	1.819	0.192
Unmethylated	219 (35.8)	103 (33.9)	105 (39.3)	11 (26.8)				
Methylated	393 (64.2)	201 (66.1)	162 (60.7)	30 (73.2)				
ABCC6, no. (%)					6.282	0.043*	6.119	0.016*
Unmethylated	523 (85.5)	250 (82.2)	239 (89.5)	34 (82.9)				
Methylated	89 (14.5)	54 (17.8)	28 (10.5)	7 (17.1)				
CDH1, no. (%)					1.054	0.590	0.116	0.809
Unmethylated	524 (85.6)	260 (85.5)	231 (86.5)	33 (80.5)				
Methylated	88 (14.4)	44 (14.5)	36 (13.5)	8 (19.5)				
THBS1, no. (%)					1.041	0.594	0.096	0.772
Unmethylated	457 (74.7)	230 (75.7)	199 (74.5)	28 (68.3)				
Methylated	155 (25.3)	74 (24.3)	68 (25.5)	13 (31.7)				
BRCA1, no. (%)					2.219	0.330	0.863	0.375
Unmethylated	504 (82.4)	256 (84.2)	217 (81.3)	31 (75.6)				
Methylated	108 (17.6)	48 (15.8)	50 (18.7)	10 (24.4)				
Presence of hypermethylation in any gene, no. (%)					8.739	0.013*	8.537	0.004*
Unmethylated	70 (11.4)	24 (7.9)	42 (15.7)	4 (9.8)				
Methylated	542 (88.6)	28 (92.1)	225 (84.3)	37 (90.2)				
Mean methylated genes		3.70 ± 2.33	2.83 ± 2.18	3.85 ± 2.35	21.900	< 0.001*	−4.431	< 0.001*
Number of methylated genes, no. (%)					20.046	< 0.001*	16.108	< 0.001*
0–2	254 (41.5)	108 (35.5)	135 (50.6)	11 (26.8)				
3–5	243 (39.7)	126 (41.4)	97 (36.3)	20 (48.8)				
6–10	115 (18.8)	70 (23.0)	35 (13.1)	10 (24.4)				
Prognostic outcomes								
^a^Overall mortality, no. (%)					0.059	0.011*	4.547	0.033*
Survive	379 (66.4)	210 (69.1)	169 (63.3)	23 (56.1)				
Death	192	94 (30.9)	98 (36.7)	18 (43.9)				
^a^Cancer-specific mortality, no. (%)					0.059	0.011*	4.547	0.033*
Survive	425 (69.4)	223 (73.4)	178 (66.7)	34 (58.5)				
Death	187 (30.6)	81 (26.6)	89 (33.3)	17 (41.5)				
^a^Intravesical recurrence, no. (%)					6.131	0.047*	2.879	0.090
No recurrence	438 (71.6)	228 (75.0)	185 (69.3)	25 (61.0)				
Recurrence	174 (28.4)	76 (25.0)	82 (30.7)	16 (39.0)				
^a^Contralateral recurrence, no. (%)					6.668	0.036*	0.610	0.435
No recurrence	580 (94.8)	291 (95.7)	253 (94.8)	41 (87.8)				
Recurrence	32 (5.2)	13 (4.3)	14 (5.2)	5 (12.2)				

*CKD* chronic kidney disease, *eGFR* estimated glomerular filtration rate, *SD* standard deviation

*Statistically significant

^a^Log-rank test was used

## Discussions

In a meta-analysis which included 17 studies with 12,094 patients, Wu et al. demonstrated that ureteral tumors exhibited worse CSS and recurrence-free survival than renal pelvic tumors based on adjusted HRs; however, no such results were noticed in subgroup analysis of pT3/4 and pN1 tumors, though the authors observed significant heterogeneity among reported articles [4]. The only corresponding study that additionally included molecular work was published in 2013, in which Krabbe et al. found no difference in the expression of p21, p27, p53, cyclin E, and Ki-67 [8].

Regarding the relatively higher stages of renal pelvic tumors, Raman et al. suggested that ureteral tumors tend to be diagnosed earlier due to ureteric obstruction, and thus were likely to be detected at a lower stage [5]. In the current cohort of patients, more patients with renal pelvic tumors were diagnosed due to gross hematuria, while the prevalent presence of hydronephrosis could help the detection of ureteral tumors by ultrasound in annual regular physical examination in many patients.

It’s interesting that the presence of sessile architecture and higher tumor grade was more common in ureteral tumors, which indicated the higher aggressiveness of ureteral tumors, as demonstrated in prior studies [4]. The change of DNA methylation status is regarded to be a key event in transcriptionally repressed regions of the genome [12]. Hypermethylation is a mechanism for repression of gene transcription in cancer [9]. Prior studies on bladder cancer demonstrated aberrant methylation status of some specific gene promoter as a sign of higher aggressiveness and worse prognosis [11, 15–19]. We similarly found that increased number of methylated genes appeared to correlate with worse CSS.

Our results demonstrate that renal pelvic and ureteral tumors, though both belong to UTUC, are not totally biologically homogenous and might behave differently. It’s interesting that the rate of hypermethylation was much more higher in renal pelvis tumors than in the ureter, but the ureteral tumors exhibited higher aggressiveness and relatively worse prognosis. What’s more, it’s notable that on sub-analysis, the number of methylated genes was a stronger driver for oncologic outcomes in ureteral tumors. This being said, however, each gene must also be viewed separately, as the prognostic effect of gene hypermethylation appeared to differ by location, further implicating differences in underlying biology between the two groups.

In a published Meta-analysis ureteral location was related to higher risk of bladder recurrence [21]. Although no statistical difference was found in our study, a more distally located tumor within the ureter could conceivably affect bladder recurrence as seen in our previous publication [22].The analysis with gene methylation status didn’t seem to be very informative for this phenomenon. In a Japanese multi-institutional study, Tanaka et al. found that the patterns of tumor spread was related to primary location of the urothelial carcinoma: patients with ureteral tumors (especially at middle and lower part) tended to suffer from local recurrence in the pelvic cavity, while renal pelvic tumors were associated with higher risk of lung metastasis [7]. The underlying biological mechanisms about the differences in the patterns of tumor metastasis corresponding to tumor location remain to be elucidated in the future.

Our study has several limitations related to the retrospective design, and there might be some selection and recall bias, especially considering some patients were excluded due to the unavailable extracted DNA for test. The exact rate and site of distant metastasis and local recurrence were also incompletely available, which precluded further analysis concerning difference patterns of disease recurrence.

Despite these limitations, our study was the first comparative study that integrated epigenetic information with UTUC tumor location, and to our knowledge, the first study that demonstrated the higher prevalence of gene promoter hyper-methylation in renal pelvic tumors. Indeed, future research is warranted to further elucidate the role that gene methylation plays in the development and biology of renal pelvic and ureteral tumors.

## Conclusion

Renal pelvic tumors and ureteral tumors exhibited significant differences in clinicopathologic characteristics and epigenetic biomarkers. Gene promoter methylation might be an important mechanism in explaining distinct tumor patterns and behaviors in UTUC.

## Abbreviations

CSS: Cancer specific survival
CT: Computed tomography
eGFR: Estimated glomerular filtration rate
HR: Hazard ratio
MRI: Magnetic resonance imaging
MSP: Methylation-sensitive polymerase chain reaction
OS: Overall survival
RNU: Radical nephroureterectomy
UICC: Union for International Cancer Control
UTUC: Upper tract urothelial carcinoma
WHO: World Health Organization
